# Anthracycline-induced cardiorenal toxicity: from molecular mechanisms to clinical management

**DOI:** 10.3389/fcvm.2026.1811848

**Published:** 2026-05-12

**Authors:** Linke Jiao, Chao Yuan, Li Wang, Yating Zhao, Guoxia Zhang, Xinyu Yang, Yonghong Gao, Fan Yang, Xinye Li, Na An, Hongcai Shang, Yanwei Xing

**Affiliations:** 1 Department of Cardiology, Guang’anmen Hospital, China Academy of Chinese Medical Sciences, Beijing, China; 2 Department of Breast Surgery, Dezhou Second People’s Hospital, Dezhou, China; 3 Department of Breast Surgery, Xingtai People’s Hospital, Xingtai, China; 4 Department of Breast Surgery, North China University of Science and Technology Affiliated Hospital, Tangshan, China; 5 Department of Critical Care Medicine, Dongzhimen Hospital, Beijing University of Chinese Medicine, Beijing, China; 6 Key Laboratory of Chinese Internal Medicine of Ministry of Education, Dongzhimen Hospital, Beijing University of Chinese Medicine, Beijing, China; 7 Institute of Acupuncture and Moxibustion, China Academy of Chinese Medical Sciences, Beijing, China; 8 Dongfang Hospital, Beijing University of Chinese Medicine, Beijing, China

**Keywords:** anthracyclines, cardiorenal toxicity, hemodynamic, molecular mechanisms, neuroendocrine, treatment strategies

## Abstract

In oncology clinics, anthracyclines remain a cornerstone of cancer therapy. However, their clinical benefits are accompanied by substantial toxicities. As cumulative exposure increases and patient survival improves, the incidence of treatment-related adverse effects also increases, particularly those affecting the heart and kidneys. Rather than existing in isolation, these two forms of damage often interact: impaired cardiac function can accelerate renal injury through hemodynamic changes and neuroendocrine activation, whereas renal dysfunction further exacerbates cardiac injury via toxin accumulation and inflammation. This interaction leads to the well-recognized cardiorenal vicious cycle, which substantially limits the safe use of anthracyclines in clinical practice. Although dexrazoxane is currently the only approved cardioprotective agent, its renoprotective effect remains limited. Moreover, there are no clear guidelines or standardized strategies for preventing or managing anthracycline-induced cardiorenal toxicity. Therefore, a deeper understanding of the underlying mechanisms, along with effective monitoring and intervention strategies, is crucial for optimizing anthracycline therapy and improving patient survival. This review explores the pathological basis, molecular mechanisms, risk factors, monitoring approaches, and treatment strategies related to anthracycline-induced cardiorenal toxicity. It aims to develop a comprehensive strategy to protect both cardiac and renal function and to provide a scientific foundation for the development of safer and more effective cancer treatment regimens.

## 1 Introduction

Anthracyclines are widely used in clinical oncology due to their potent antitumor efficacy. Despite the rapid development of immunotherapy and targeted therapies, anthracyclines remain a cornerstone for the treatment of malignancies such as breast cancer, lymphoma, and leukemia (1, 2). Commonly used agents include doxorubicin (DOX), daunorubicin, and epirubicin. However, while exerting their antitumor effects, anthracyclines are frequently associated with severe adverse events, including cardiotoxicity, nephrotoxicity, and myelosuppression, making their clinical use a double-edged sword (3). These toxicities primarily result from the lack of tissue specificity of anthracyclines, leading to their accumulation in normal tissues and subsequent damage in addition to their effects on tumor cells (3, 4). Among these adverse effects, cardiotoxicity represents the major limiting factor in anthracycline therapy, with an incidence ranging from 5% to 48% and a clear dose-dependent pattern (5). The clinical manifestations are highly heterogeneous, ranging from asymptomatic cardiac dysfunction to irreversible myocardial injury and death (6–8). It has been reported that approximately 2%–4% of affected patients progress to congestive heart failure, 9%–11% exhibit subclinical structural changes, more than 12% develop arrhythmias, and 30%–35% show elevated cardiac biomarkers of injury (9). In addition, there is substantial interindividual variability in susceptibility to anthracyclines, with some patients tolerating standard doses without long-term complications, whereas others may develop cardiotoxicity even after the first administration (5).

Beyond cardiac injury, accumulating evidence indicates that anthracyclines can also induce renal damage. Experimental and clinical studies have shown that anthracycline-induced nephrotoxicity is characterized by disruption of the glomerular filtration barrier, tubular dilation, epithelial cell degeneration, inflammatory cell infiltration, and interstitial fibrosis. Clinically, this manifests as elevated serum creatinine and blood urea nitrogen levels, proteinuria, hypoalbuminemia, and edema (10–12). Taking DOX as an example, it is recognized as an independent risk factor for chemotherapy-related renal injury, with renal adverse events occurring in approximately 12%–18% of patients receiving DOX-containing regimens (13). Epidemiological studies further suggest that anthracycline exposure, particularly at high cumulative doses, is associated with an increased risk of late-onset renal failure (14) and may contribute to the development and progression of acute kidney injury (AKI) and chronic kidney disease (CKD) (15). However, large-scale epidemiological studies focusing on renal toxicity as a primary endpoint remain limited, as most existing studies emphasize cardiotoxicity or mechanistic investigations. Therefore, the precise incidence of anthracycline-induced nephrotoxicity remains uncertain. Importantly, anthracycline-induced renal injury does not arise from a single mechanism but results from the combined effects of direct nephrotoxicity and secondary consequences of cardiotoxicity. On the one hand, anthracyclines can directly damage renal tubular epithelial cells through mechanisms such as oxidative stress, mitochondrial dysfunction, and inflammatory responses. On the other hand, anthracycline-induced cardiac dysfunction may exacerbate renal injury by reducing cardiac output, impairing renal perfusion, and activating neurohumoral pathways, thereby representing a typical cardiorenal interaction.

Given the close anatomical and physiological relationship between the heart and kidneys, these organs exhibit strong bidirectional interactions under pathological conditions. Patients with pre-existing cardiac dysfunction are more susceptible to renal injury during anthracycline therapy, while renal impairment, in turn, exacerbates cardiovascular burden (16). Similarly, baseline renal dysfunction significantly increases the risk of anthracycline-induced cardiotoxicity and further accelerates the deterioration of both cardiac and renal function, ultimately exacerbating the overall disease burden (17, 18). This vicious cycle is particularly pronounced in elderly patients and those with comorbidities, who are at higher risk of multi-organ damage. Therefore, whether occurring independently or concurrently, anthracycline-associated cardiorenal toxicity warrants substantial clinical attention. However, standardized strategies for monitoring and managing these toxicities remain lacking. This not only limits the safe use of anthracyclines but also highlights the urgent need to elucidate the underlying mechanisms and develop effective preventive and therapeutic approaches. In this context, the present review systematically summarizes the pathological basis and molecular mechanisms of anthracycline-induced cardiotoxicity and nephrotoxicity, as well as their associated risk factors, monitoring strategies, and therapeutic interventions. It aims to provide a theoretical foundation for optimizing clinical management and achieving a balance between therapeutic efficacy and long-term safety.

## 2 Pathophysiology of anthracycline-induced cardiorenal toxicity

The importance of anthracycline-induced cardiorenal toxicity warrants particular attention. This is primarily attributed to the tightly linked physiology of the heart and kidneys. The following sections outline the key pathophysiological pathways underlying this coupled toxicity.

### 2.1 Hemodynamic homeostasis

**Figure 1:**
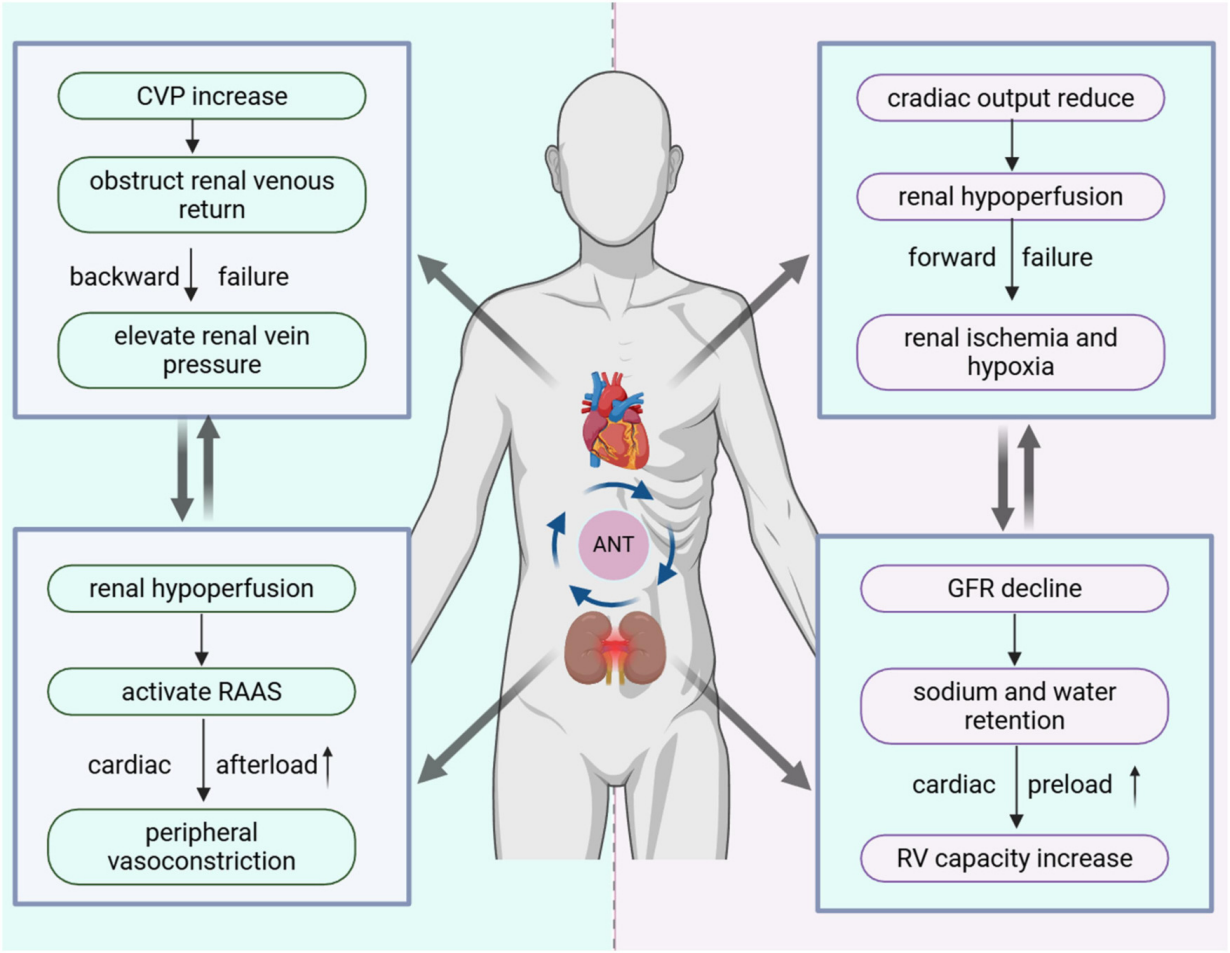
Effects of hemodynamic changes on anthracycline-induced cardiorenal toxicity. Anthracycline-induced cardiotoxicity can impair renal function by reducing cardiac output, leading to inadequate renal perfusion or elevated renal venous pressure. In turn, anthracycline-induced nephrotoxicity can exacerbate cardiac preload and afterload due to renal insufficiency, thereby creating a vicious cycle between cardiac and renal function. ANT, anthracyclines; CVP, central venous pressure; RAAS, renin–angiotensin–aldosterone system; RV, right ventricle.

Under normal physiological conditions, the heart and kidneys function in a tightly coordinated partnership, forming a dynamic feedback system essential for hemodynamic stability and systemic balance (19). Each cardiac contraction propels blood into the arterial circulation, maintaining adequate perfusion pressure throughout the body. In return, the kidneys receive roughly one-fifth of the cardiac output, a volume crucial for sustaining glomerular filtration pressure and enabling efficient clearance of metabolic waste. However, this interaction extends beyond these processes. Through sodium and water reabsorption, the kidneys dynamically adjust circulating blood volume, directly influencing cardiac preload and output. They also contribute to electrolyte homeostasis, which subsequently influences myocardial electrophysiology (16, 19). Through this bidirectional feedback mechanism involving cardiac pumping, renal filtration, and volume regulation, the heart and kidneys form a central circuit that maintains systemic hemodynamic stability and internal homeostasis. When anthracyclines disrupt one component of this circuit, the other is often affected as well. This provides a plausible explanation for the frequently observed synergistic pattern of cardiorenal injury in clinical practice (Figure 1).

When anthracycline-induced cardiotoxicity leads to hemodynamic disturbances, renal function is primarily affected through two mechanisms. The first stems from left ventricular dysfunction. A decline in cardiac output leads to significant renal hypoperfusion. Initially, renal compensatory mechanisms, including tubuloglomerular feedback and activation of the renin-angiotensin-aldosterone system (RAAS), help preserve intraglomerular pressure and renal blood flow (20). With progressive heart failure, however, the sustained drop in perfusion eventually overwhelms these adaptations. This forward failure state, driven by prolonged hypoperfusion, leads to renal tubular ischemia, hypoxia, and subsequent kidney injury (21–23). The second pathway arises from right heart failure. Increased cardiac volume load elevates central venous pressure (CVP), which impedes renal venous return. The consequent rise in renal venous pressure, often referred to as backward failure, increases renal afterload, compresses the renal interstitium and capsule, and ultimately reduces the glomerular filtration rate (GFR) (23, 24). These mechanisms are well supported by clinical evidence. Studies have shown that patients receiving anthracycline therapy face a substantially higher risk of cardiac damage than those who do not (25, 26), and such injury directly impairs renal perfusion. This association was recognized decades ago. An early DOX-induced rabbit model from the 1980s demonstrated that low-output congestive heart failure consistently correlated with a pronounced reduction in renal blood flow (27). That landmark study first illustrated how anthracycline-induced hemodynamic alterations can simultaneously disrupt both cardiac and renal function.

The interplay between the heart and kidneys is fundamentally bidirectional. When anthracycline-induced nephrotoxicity occurs, the resulting hemodynamic disturbances further exacerbate cardiac strain through two interrelated pathways. On the one hand, sodium and water retention caused by declining renal function increases circulating blood volume. This leads to right ventricular (RV) dilation, increases cardiac preload, and gradually impairs myocardial efficiency (28, 29). On the other hand, renal hypoperfusion activates RAAS, triggering peripheral vasoconstriction. This impairs cardiac function and increases cardiac afterload (30, 31). Clinically, patients with renal insufficiency face a markedly higher risk of cardiotoxicity, particularly heart failure, during chemotherapy. Experimental models further support this interaction. Data indicate that combining DOX exposure with renal ischemia inflicts greater cardiac damage than either condition alone. The combined injury manifests as a reduced left ventricular ejection fraction (LVEF), accompanied by an intensified inflammatory response, cardiomyocyte apoptosis, fibrosis, and vacuolar degeneration (17). Notably, in models with pre-existing cardiomyopathy, cardiac injury induced by renal impairment is significantly amplified, confirming that renal dysfunction exacerbates existing heart disease. The study also highlighted a synergistic effect between DOX-induced renal ischemia-reperfusion injury and cardiac dysfunction, with fluid retention implicated throughout this pathological process (17).

In summary, when anthracycline-induced toxicity affects either organ, the heart and kidneys interact and may form a vicious cycle of dysfunction, with hemodynamic alterations serving as a key driver of this pathological cascade.

### 2.2 Neuroendocrine activation

**Figure 2:**
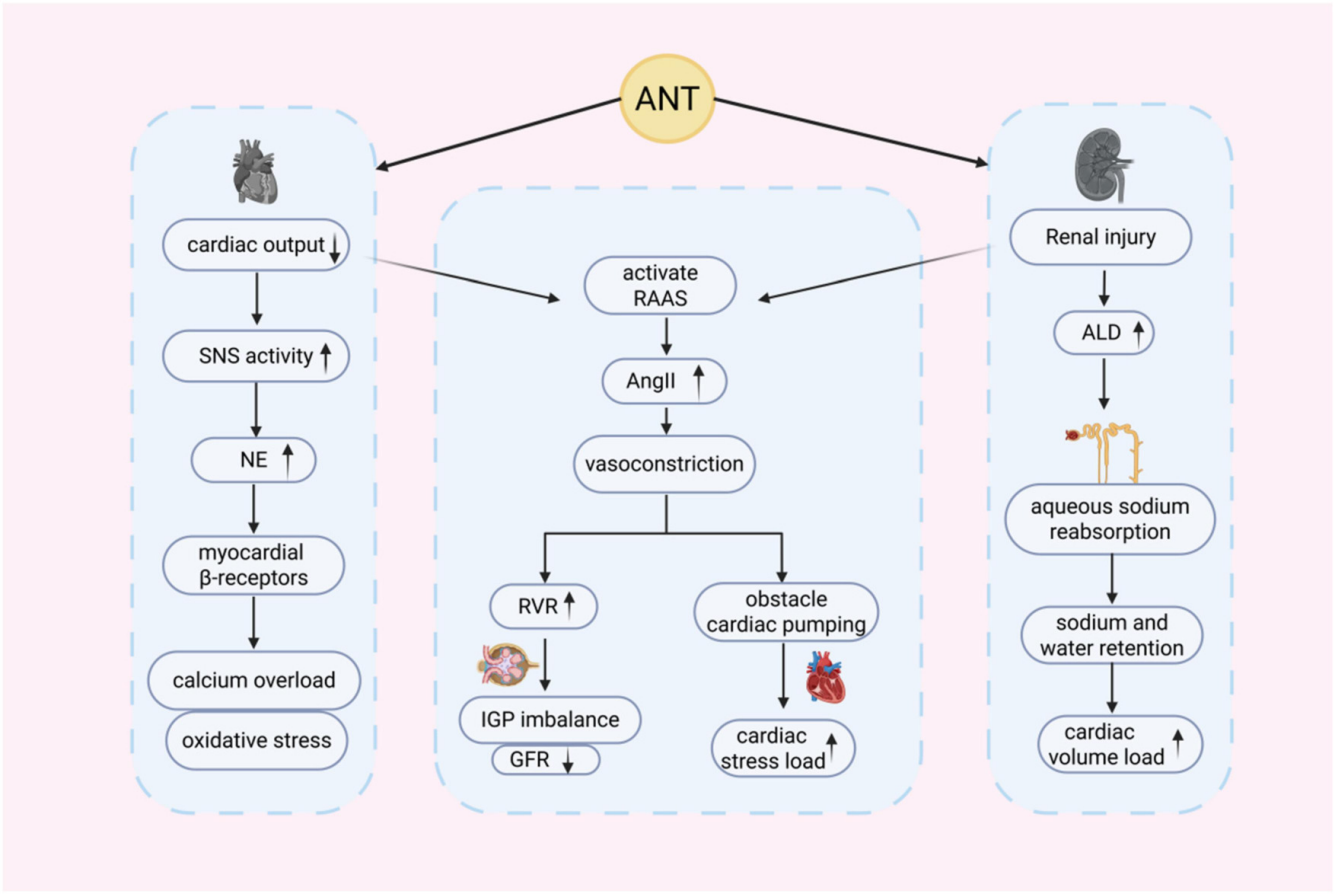
Effects of neuroendocrine dysregulation on anthracycline-induced cardiorenal toxicity. Anthracyclines impair cardiac and renal function by aberrantly activating the RAAS and SNS signaling cascades. ANT, anthracyclines; SNS, sympathetic nervous system; NE, norepinephrine; ALD, aldosterone; RVR, renal vascular resistance.

Beyond hemodynamic interactions, the heart and kidneys communicate through shared neuroendocrine pathways, primarily involving the sympathetic nervous system (SNS), RAAS, and the natriuretic peptide system. Together, these systems coordinate a multilayered hormonal response that adjusts cardiac output, renal perfusion, and fluid balance. When cardiac output declines, the SNS is rapidly activated. Its activation increases norepinephrine (NE) release, thereby restoring cardiac output by elevating heart rate and contractility. Simultaneously, sympathetic activation constricts renal vessels and stimulates renin secretion, initiating a pressor response to support systemic blood pressure. The consequent reduction in effective circulating volume further activates RAAS, stimulating aldosterone (ALD) secretion. This promotes sodium and water reabsorption in renal tubules and collecting ducts, thereby increasing blood volume and cardiac preload as a compensatory mechanism to regulate fluid, electrolyte, and acid-base balance (32). Meanwhile, the heart releases natriuretic peptides to counteract these signals. In conditions like congestive heart failure, elevated atrial pressure stimulates the secretion of atrial natriuretic peptide (ANP) and brain natriuretic peptide (BNP). These peptides antagonize the fluid-retentive effects driven by SNS and RAAS, promoting renal vasodilation and enhancing water and sodium excretion (33). Under physiological conditions, these interconnected neuroendocrine systems maintain functional equilibrium within the cardiorenal system through bidirectional feedback. However, under anthracycline-induced toxicity, this delicate balance is often disrupted, thereby accelerating a vicious cycle of mutual organ injury (Figure 2).

RAAS serves as a key regulator of blood pressure and fluid balance, as well as a critical neuroendocrine pathway in cardiorenal interactions. Its dysregulation is implicated in a spectrum of disease states (34). Inappropriate RAAS activation appears to be a major driver of cardiac and renal dysfunction associated with anthracycline therapy. Studies suggest that anthracyclines can directly affect RAAS activity. In mouse models, DOX treatment significantly elevates levels of angiotensin-converting enzyme (ACE), along with plasma renin, angiotensin II (Ang II), and ALD (35). This initiates a well-characterized yet deleterious cascade. Under pathological conditions, renin cleaves liver-derived angiotensinogen to form Ang I, which ACE then converts to the potent effector Ang II. Ang II in turn stimulates ALD secretion, promoting sodium and water reabsorption while triggering vasoconstriction. The combined effects result in increased blood pressure and volume overload, thereby imposing significant stress on both cardiac and renal tissues (35, 36). In anthracycline-induced heart failure, reduced cardiac output leads to decreased renal perfusion, thereby activating RAAS. Ang II mediates vasoconstriction and increases renal vascular resistance (RVR), exacerbating glomerular ischemia and hypoxia. It also impairs cardiac function and elevates cardiac pressure load, further worsening heart failure (37, 38). ALD promotes sodium and water reabsorption in the distal renal tubules and collecting ducts, resulting in fluid retention and increased cardiac preload, which further compromises cardiac function (39). In DOX-induced renal injury, this agent disrupts RAAS homeostasis, leading to excessive Ang II production and impaired regulation of intraglomerular pressure, thereby causing interstitial injury and renal edema. At the same time, Ang II stimulates NADPH oxidase to produce excessive reactive oxygen species (ROS), thereby accelerating inflammation and fibrosis (4). Animal studies further demonstrate that DOX induces endogenous activation of RAAS, contributing to cardiorenal dysfunction. In the early stage, compensatory RAAS activation may not immediately result in overt renal impairment. However, with prolonged exposure, sustained overactivation ultimately leads to progressive cardiorenal injury (40, 41). Moreover, ACE inhibitors (ACEIs) have been shown to significantly attenuate DOX-induced cardiorenal toxicity, further supporting a central role of RAAS in anthracycline-induced cardiorenal injury (42). This therapeutic evidence not only substantiates the central role of RAAS in anthracycline-related cardiorenal injury but also highlights its potential as a therapeutic target.

Natriuretic peptides function as a key physiological counter-regulatory system that mitigates volume overload and neuroendocrine activation. By promoting natriuresis and diuresis, dilating blood vessels, and lowering blood pressure, they effectively reduce total circulating blood volume. Concurrently, they confer direct cardiorenal protection by antagonizing RAAS overactivation and inhibiting cellular apoptosis and fibrosis (43). However, during the decompensated phase of cardiac and renal dysfunction following anthracycline chemotherapy, circulating natriuretic peptide levels increase in proportion to the severity of organ injury. Serum NT-proBNP levels are significantly elevated in patients with cardiotoxicity. Notably, patients with renal impairment also exhibit increased NT-proBNP levels, which are independently associated with heightened cardiovascular risk (44, 45). This clinical pattern is consistently replicated in preclinical models. Experimental studies have shown that DOX-treated animal models exhibit significantly elevated serum NT-proBNP levels alongside marked structural damage, including reduced cardiomyocyte cross-sectional area and exacerbated myocardial fibrosis (46). Further studies in mouse and rabbit models confirm that DOX upregulates key natriuretic peptides, including ANP and BNP, which correlate with measurable declines in both cardiac and renal function (47, 48).

Autonomic dysfunction has been reported in some cancer patients and may exacerbate chemotherapy-induced cardiac and renal injury (49). Studies have shown that anthracyclines can directly enhance SNS activity. DOX-treated cardiac tissue exhibits sympathetic nerve fiber remodeling and elevated plasma NE levels. These changes may lead to left ventricular dysfunction and even heart failure, whereas sympatholytic agents can effectively protect the heart from anthracycline-induced injury (50, 51). A similar pattern is observed in renal tissue. In spontaneously hypertensive rats treated with DOX, increased SNS activity correlates with elevated urinary corticosterone, albumin excretion, and NE. Histologically, these changes coincide with glomerulosclerosis and tubulointerstitial damage (52). The core pathological mechanism is the dual damaging effects of NE released through SNS overactivation on the heart and kidneys (53). In the heart, NE overstimulates myocardial β-receptors, triggering calcium overload and oxidative stress, which accelerates cardiomyocyte apoptosis and fibrosis. Simultaneously, NE activates RAAS, increasing both cardiac preload and afterload and further compromising cardiac function. In the kidneys, NE induces vasoconstriction, reducing perfusion and exacerbating ischemic tubular necrosis. It also stimulates renin release from juxtaglomerular cells, thereby amplifying RAAS activation and promoting sodium retention and interstitial fibrosis, ultimately leading to a decline in GFR (19). Furthermore, SNS and RAAS engage in a vicious cycle of mutual activation. NE promotes excessive RAAS activation, and Ang II downstream of RAAS in turn further enhances SNS activity and prolongs NE action. This bidirectional amplification of cardiac and renal injury ultimately progresses from single-organ dysfunction to a mutually reinforcing deterioration of cardiorenal function (54).

The heart and kidneys are physiologically coupled, a relationship that predisposes both organs to bidirectional injury under pathological stress. Anthracycline-induced injury to either organ can propagate to the other through two interconnected pathways: hemodynamic disturbance and neuroendocrine dysregulation. These mechanisms not only coexist but also interact synergistically, accelerating a vicious cycle of mutual functional decline. This pathophysiology underscores the intrinsic link between cardiac and renal damage and supports an integrated clinical approach. Consequently, during anthracycline chemotherapy, a combined cardiorenal monitoring system should be implemented alongside comprehensive management strategies. The goal is to interrupt the cascade triggered by single-organ toxicity at its source, thereby providing safer and more reliable protection for patients during treatment.

## 3 Molecular mechanisms of anthracycline-induced cardiorenal toxicity

The molecular mechanisms underlying anthracycline-induced cardiorenal toxicity remain incompletely understood. The sequence in which cardiotoxicity and nephrotoxicity occur has not yet been fully clarified. Some studies suggest that anthracycline-induced cardiotoxicity may indirectly cause kidney injury by altering renal hemodynamics and disrupting renal metabolic function (55), whereas other evidence indicates that nephrotoxicity may precede cardiotoxicity. For example, toxin retention and the release of inflammatory mediators caused by anthracycline accumulation in the kidneys may, in turn, exacerbate myocardial injury (56, 57). Regardless of the initiating sequence, the close physiological connection between the heart and kidneys predisposes them to a synergistic and mutually aggravating injury. An initial insult to one organ can swiftly propagate to the other through shared hemodynamic and neuroendocrine pathways, establishing a vicious cycle that is difficult to interrupt. This synergistic injury not only compromises the structure and function of both organs but also increases the likelihood of unplanned chemotherapy modifications, ultimately affecting treatment outcomes.

**Figure 3:**
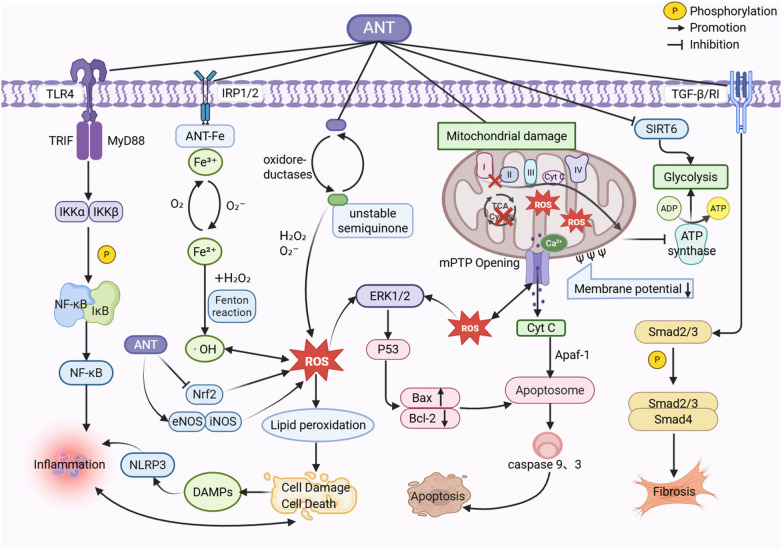
Molecular mechanisms of anthracycline-induced cardiorenal toxicity. During semiquinone formation and iron chelation, anthracyclines generate various free radicals, leading to severe oxidative damage in cardiac and renal tissues. Concurrently, elevated ROS levels exacerbate inflammatory responses, mitochondrial dysfunction, and apoptosis, thereby creating an interconnected and mutually reinforcing network of pathological mechanisms. Furthermore, anthracyclines activate key inflammatory mediators, including NF-κB and NLRP3, which induce inflammatory injury in both cardiac and renal tissues. Anthracyclines also directly damage mitochondrial structure, inhibit the tricarboxylic acid cycle, redirect metabolism toward glycolysis, impair adenosine triphosphate (ATP) synthesis, and initiate the intrinsic apoptotic signaling pathway. Additionally, anthracyclines upregulate the expression of TGF-β, increase collagen deposition, and thereby contribute to cardiac and renal injury. ANT, anthracyclines.

Accumulating evidence indicates that the development and progression of anthracycline-induced cardiorenal toxicity are closely associated with multiple molecular mechanisms, among which oxidative stress, inflammation, mitochondrial dysfunction, and apoptosis constitute the core pathways. These mechanisms do not act in isolation but are highly interconnected and mutually reinforcing, ultimately leading to structural damage and functional impairment of both the heart and kidneys (Figure 3) (55, 58). With advances in molecular biology, our understanding of these shared mechanisms has continued to evolve. From anthracycline-triggered ROS overproduction to the activation of inflammatory pathways, from mitochondrial injury to the initiation of apoptotic cascades, and ultimately to transforming growth factor-β (TGF-β)-mediated fibrosis, a growing body of evidence has progressively elucidated the pathological basis of anthracycline-induced cardiorenal toxicity. These insights provide a strong theoretical foundation for the development of targeted preventive and therapeutic strategies, as well as for improving the safety of anthracycline-based therapies. The following sections further explore these core mechanistic dimensions, aiming to provide a foundation for the development of integrated cardiorenal protective strategies.

### 3.1 Oxidative stress

Anthracyclines induce oxidative injury in cardiorenal tissues through multiple convergent pathways, overwhelming endogenous antioxidant defenses and disrupting redox homeostasis (59–62). This effect is not cell-type-specific and affects both malignant and normal cells (63). Extensive evidence indicates that anthracyclines downregulate key antioxidant enzymes, including superoxide dismutase (SOD), catalase (CAT), and glutathione peroxidase (GPx), while increasing levels of malondialdehyde (MDA), a marker of lipid peroxidation. This coordinated disruption impairs the cellular antioxidant defense system, ultimately leading to structural and functional damage in both the heart and kidneys (60, 64, 65). At the molecular level, the quinone moiety of anthracyclines undergoes one-electron reduction by oxidoreductases such as NADPH oxidoreductase and xanthine oxidase, generating an unstable semiquinone radical. This radical rapidly transfers an electron to molecular oxygen (O₂), regenerating the parent quinone and producing a superoxide anion (O₂⁻). Superoxide dismutase subsequently converts O₂⁻ into hydrogen peroxide (H₂O₂), thereby sustaining a redox cycling process that continuously generates ROS (66, 67). The resulting ROS overload promotes lipid peroxidation, damages membranes and DNA, and triggers apoptotic pathways. Furthermore, anthracyclines can directly chelate iron to form complexes, further amplifying oxidative damage. Superoxide reduces Fe^3^⁺ in the complex to Fe^2^⁺, which then reacts with H₂O₂ via the Fenton reaction to generate highly toxic hydroxyl radicals (·OH). Residual Fe^2^⁺ is re-oxidized to Fe^3^⁺ by O₂, continuously supplying substrates for the Fenton reaction and dramatically enhancing oxidative stress (68). Concurrently, anthracycline-mediated iron dysregulation involves iron regulatory proteins (IRP 1/2) that bind to iron response elements (IREs). This interaction suppresses ferritin synthesis while upregulating transferrin receptor (TfR) translation, increasing iron uptake and further promoting ROS generation and iron-dependent apoptosis (63, 68–71). Notably, this oxidative burst serves as a key upstream trigger. ROS activate transcription factors such as NF-κB, a master regulator of inflammation, immune responses, and cell survival, thereby promoting the expression of pro-inflammatory cytokines. Simultaneously, ROS disrupt mitochondrial integrity and initiate intrinsic apoptotic pathways (60, 72).

Nuclear factor erythroid 2-related factor 2 (Nrf2) is a pivotal regulator of cellular redox homeostasis. Under basal conditions, Nrf2 is sequestered in the cytoplasm by its repressor protein, Kelch-like ECH-associated protein 1 (Keap1) (73). Cellular stress triggers the dissociation of this complex, allowing Nrf2 to translocate to the nucleus, where it binds to antioxidant response elements (AREs) and induces the transcription of cytoprotective enzymes, including superoxide dismutase (SOD), catalase (CAT), and heme oxygenase-1 (HO-1) (74, 75). In models of anthracycline-induced cardiorenal toxicity, this crucial defensive axis is impaired. Nrf2 expression is significantly downregulated, accompanied by reduced levels of its downstream effectors such as HO-1. This impairment of the antioxidant response renders cardiac and renal tissues more susceptible to oxidative damage (66, 76, 77). In addition to ROS-mediated oxidative stress, anthracyclines also induce nitrosative stress mediated by reactive nitrogen species (RNS). RNS are highly reactive oxidants primarily generated through the interaction of nitric oxide (NO) with superoxide, among other pathways. Accordingly, intracellular NO availability is a key determinant of nitrosative stress. NO is synthesized by nitric oxide synthase (NOS), which comprises three primary isoforms: neuronal (nNOS), inducible (iNOS), and endothelial (eNOS). In cardiorenal tissues, iNOS and eNOS isoforms are the predominant sources of NO production (66). Studies indicate that anthracyclines upregulate iNOS and eNOS expression in cardiac and renal tissues, leading to excessive NO generation. Meanwhile, elevated levels of H₂O₂ and O₂⁻ further amplify oxidative stress and interact with NO to enhance nitrosative stress, thereby exacerbating anthracycline-induced cardiorenal injury (66, 75, 78).

### 3.2 Inflammatory response

Inflammation represents another core mechanism underlying anthracycline-induced cardiorenal toxicity. This process is primarily driven by activation of the NF-κB signaling pathway and assembly of the NOD-like receptor protein 3 (NLRP3) inflammasome, which together form a central regulatory network. These pathways promote the release of pro-inflammatory cytokines and the infiltration of inflammatory cells, ultimately contributing to cardiorenal injury.

The NF-κB signaling pathway is activated through two main branches: canonical and non-canonical. The canonical pathway responds rapidly to tissue injury or stimulation, including microbial components, pro-inflammatory cytokines such as tumor necrosis factor-α (TNF-α) and interleukin-1 (IL-1), and Toll-like receptor (TLR) engagement (79). Under basal conditions, NF-κB is sequestered in the cytoplasm in an inactive complex with its inhibitor, the inhibitor of NF-κB (IκB). Anthracycline exposure, however, induces ROS accumulation in cardiac and renal tissues and activates TLR4, initiating a phosphorylation cascade that involves IκB kinase (IKK) and NF-κB p65 (80, 81). TLR4 signals through adaptor proteins such as MyD88 or TRIF to activate the IKK complex, which comprises the catalytic subunits IKKα and IKKβ and the regulatory subunit IKKγ (also known as NEMO) (82). Activated IKK phosphorylates IκB, targeting it for ubiquitination and degradation. This releases NF-κB, allowing it to translocate to the nucleus and promote the transcription of target genes that amplify the inflammatory response (83). Anthracyclines can further intensify this signaling through parallel pathways, including lysophosphatidic acid receptor 1 (LPAR1), ERK1/2, and p38 mitogen-activated protein kinase (MAPK), collectively promoting progressive inflammatory injury in cardiorenal tissues (84, 85). Notably, anthracycline-induced oxidative stress and cellular damage lead to the release of damage-associated molecular patterns (DAMPs). These endogenous signals synergize with TLR-mediated activation to upregulate NLRP3 transcription (86). As a key component of the innate immune system, the assembled NLRP3 inflammasome activates caspase-1, which promotes the maturation and secretion of pro-inflammatory cytokines, including IL-1β and IL-18, thereby amplifying the inflammatory cascade in cardiorenal tissues (86, 87).

Following the coordinated activation of the NF-κB pathway and NLRP3 inflammasome, cardiorenal tissues exhibit marked upregulation of pro-inflammatory cytokines, resulting in a cytokine surge. Experimental evidence indicates that anthracycline exposure elevates levels of inflammatory mediators, including TNF-α, IL-6, IL-1β, IL-33, and tumor necrosis factor receptor (TNFR), while also increasing the mRNA expression of kidney injury molecule-1 (KIM-1). These molecules directly damage cardiomyocytes, renal tubular epithelial cells, and podocytes, promoting apoptosis and functional impairment (88–91). TNF-α acts as a central toxic mediator in this cascade. In the heart, it recruits neutrophils and monocytes and induces cardiomyocyte hypertrophy and fibrosis, thereby reducing cardiac contractile function. In the kidney, it drives glomerular hypertrophy, mesangial matrix expansion, and basement membrane thickening—changes that ultimately contribute to proteinuria, glomerulosclerosis, and renal tubulointerstitial fibrosis (66). Neutrophils serve as early responders in this inflammatory milieu. Within cardiac tissue, they infiltrate and exacerbate local oxidative and inflammatory injury by releasing chemokines, matrix metalloproteinase-9 (MMP9), and myeloperoxidase (MPO) (92). Clinical studies have shown that in patients receiving DOX chemotherapy, levels of neutrophil-associated markers—peptidoglycan recognition protein 1, MMP9, and MPO—in peripheral blood are significantly increased and correlate closely with cardiotoxicity risk (92). In the kidney, although direct neutrophil infiltration may be less prominent, DOX contributes to injury partly through the release of neutrophil gelatinase-associated lipocalin (NGAL). As a neutrophil-associated injury marker, plasma NGAL levels increase significantly in DOX-induced renal damage (93). Moreover, DOX-induced NF-κB activation stimulates the release of TNF-α and IL-6, which in turn amplifies neutrophil-related inflammatory cascades. This interplay between neutrophil-derived toxic mediators and DOX-induced oxidative stress disrupts the renal tubular basement membrane, accelerates glomerulosclerosis and interstitial fibrosis, and ultimately exacerbates renal injury (93).

### 3.3 Mitochondrial damage

Mitochondria are the primary sites of cellular energy production and metabolic regulation, generating ATP through oxidative phosphorylation (OXPHOS). As key organelles supporting energy-demanding organs such as the heart and kidneys, mitochondrial dysfunction is closely associated with the onset and progression of cardiovascular disease and CKD (94, 95). However, while exerting antitumor effects, anthracyclines can induce cardiorenal injury by damaging mitochondrial structure and disrupting metabolic processes and mitochondrial quality control (96, 97).

Anthracyclines inflict direct structural and functional damage on mitochondria. They bind to mitochondrial DNA (mtDNA) and impair complexes I and III of the electron transport chain (ETC), thereby compromising OXPHOS and reducing ATP production (98–100). Simultaneously, they inhibit the phosphorylation of AMP-activated protein kinase (AMPK), a key cellular energy sensor, thereby impairing its ability to detect ATP deficiency and initiate compensatory responses. This ultimately results in insufficient energy supply in cardiorenal tissues, leading to impaired myocardial contractility and renal dysfunction (101–103). The oxidative stress induced by anthracyclines further compromises mitochondrial integrity. ROS oxidize proteins and lipids, damage mitochondrial DNA, and destabilize the inner mitochondrial membrane. This leads to a decline in membrane potential, triggering the opening of the mitochondrial permeability transition pore (mPTP). The subsequent release of cytochrome c and other pro-apoptotic factors initiates apoptosis. Notably, this membrane damage also increases mitochondrial ROS release, creating a vicious cycle between ROS accumulation and mitochondrial damage (104). Mitochondrial dysfunction drives metabolic reprogramming in cardiac and renal cells, shifting metabolism from the efficient tricarboxylic acid cycle and OXPHOS toward glycolysis (105). Histone deacetylase SIRT6 is an NAD⁺-dependent deacetylase. In cardiomyocytes, anthracyclines downregulate SIRT6 expression, relieving its inhibitory effects on glycolytic enzymes. This triggers a surge in glycolysis and lactate accumulation, leading to metabolic acidosis and exacerbating myocardial contractile dysfunction (106). In renal cells, the shift from OXPHOS to glycolysis reduces ATP production in glomerular cells, thereby impairing filtration function and promoting inflammatory infiltration, apoptosis, and pyknosis in renal tissues (96, 107). In addition to metabolic reprogramming, anthracyclines also inhibit mitophagy and mitochondrial biogenesis, leading to the progressive accumulation of damaged mitochondria. This is accompanied by reduced mtDNA copy number, decreased membrane potential, and impaired ATP synthesis, which exacerbates oxidative stress and apoptotic signaling (108–110). In summary, the combined disruption of energy metabolism and mitochondrial membrane integrity constitutes the core pathological basis of mitochondrial dysfunction in anthracycline-induced cardiorenal toxicity.

Mitochondrial injury rarely occurs in isolation; rather, it interacts with other pathological mechanisms to drive anthracycline-induced cardiorenal toxicity. As the primary cellular source of ROS, dysfunctional mitochondria—particularly those with impaired ETC activity—generate excessive ROS. Concurrently, this oxidative burden is further amplified by anthracyclines-iron complexes, which generate highly reactive hydroxyl radicals via the Fenton reaction. These radicals exacerbate mitochondrial lipid peroxidation, protein oxidation, and mtDNA damage, thereby accelerating the decline in mitochondrial function (104, 111). In addition, damage-associated molecular patterns (DAMPs) released from injured mitochondria can activate intracellular pattern recognition receptors, leading to NLRP3 inflammasome assembly and promoting the release of pro-inflammatory cytokines such as IL-1β and IL-18. Simultaneously, impaired mitophagy leads to the accumulation of damaged mitochondria, which continuously release DAMPs and further amplify the inflammatory cascade (86, 112). These processes establish a self-reinforcing circuit: mitochondrial damage triggers inflammation, which in turn promotes further mitochondrial and tissue injury, thereby linking oxidative stress, inflammation, and mitochondrial dysfunction into a unified pathological cascade.

### 3.4 Apoptosis

Apoptosis is a tightly regulated form of programmed cell death, essential for maintaining tissue homeostasis, clearing damaged cells, and supporting immune function. Depending on the initiating signals, apoptosis can be triggered through several major pathways: the intrinsic (mitochondrial) pathway, the extrinsic (death receptor) pathway, and the endoplasmic reticulum stress pathway. In anthracycline-induced cardiorenal toxicity, the intrinsic pathway—activated by oxidative stress, DNA damage, and direct organellar injury—plays a central role (113–115). Mitochondria serve as crucial intracellular reservoirs and buffers for Ca^2^⁺, with the mitochondrial permeability transition pore (mPTP) playing a central regulatory role. As a non-specific channel, mPTP opening is influenced by Ca^2^⁺ concentration, membrane potential, and redox state (116). Following anthracycline-induced damage, loss of mitochondrial membrane potential and calcium influx through the mitochondrial calcium uniporter promote sustained mPTP opening (108, 117). The resulting increase in membrane permeability releases pro-apoptotic factors such as cytochrome c and apoptosis-inducing factor (AIF) from the intermembrane space into the cytosol (118). Cytochrome c then binds to apoptotic protease-activating factor 1 (Apaf-1), forming the apoptosome. This complex recruits and activates initiator caspase-9, which in turn cleaves and activates executioner caspase-3, thereby triggering the apoptotic proteolytic cascade (114, 119). The balance between cell survival and death is tightly regulated by the Bcl-2 protein family, which governs interactions between anti-apoptotic (e.g., Bcl-2, Bcl-xL) and pro-apoptotic (e.g., Bax, Bak) members to determine cellular fate (120, 121). In models of DOX-induced injury, this balance is consistently shifted toward apoptosis. Cardiomyocytes, renal tubular epithelial cells, and podocytes exhibit downregulation of Bcl-2, upregulation of Bax, loss of mitochondrial membrane potential, cytochrome c release, and subsequent activation of the caspase cascade (122–124).

The upregulation of pro-apoptotic proteins is largely mediated by activation of the tumor suppressor p53. DOX-induced DNA damage markedly increases p53 expression, which subsequently upregulates downstream pro-apoptotic targets and drives cells toward apoptosis (118). As a central guardian of genomic integrity, p53 maintains cellular stability by regulating the cell cycle, DNA repair, and programmed cell death. Upon activation in response to DNA damage, p53 functions as a transcription factor that induces pro-apoptotic genes and promotes either apoptosis or cell cycle arrest (125). The MAPK cascade, a conserved intracellular signaling network, further modulates this apoptotic response. MAPK pathways react to diverse extracellular cues, including growth factors, stress signals, and cytokines, and play a context-dependent role in regulating cell survival and death. Key subfamilies involved are extracellular signal-regulated kinase (ERK1/2), c-Jun N-terminal kinase (JNK), and p38 MAPK (126). Although ERK1/2 activation is often associated with anti-apoptotic and proliferative functions—downregulating pro-apoptotic proteins and enhancing anti-apoptotic ones through transcriptional and post-translational mechanisms—it can also promote apoptosis under certain conditions (127). One important mechanism involves ERK-mediated upregulation of p53 (128). Activated p53 in turn transactivates Bcl-2 family members, promoting mitochondrial outer membrane permeabilization and initiating the intrinsic apoptotic cascade (129, 130). Preclinical studies confirm that DOX activates the MAPK pathway in cardiac and renal tissues, leading to elevated ERK1/2 and p53, reduced Bcl-2, and increased levels of Bax, caspase-3, and caspase-9 (4, 131–133). Together, these findings illustrate how the p53 and MAPK signaling axes converge to drive apoptosis in anthracycline-induced cardiorenal injury.

In addition to the intrinsic pathway, anthracyclines can also initiate apoptosis via the extrinsic, death receptor-mediated route. This pathway is activated when death ligands bind to their corresponding cell surface receptors, triggering a caspase cascade involving caspase-8 and caspase-3, ultimately leading to apoptosis (119). Key death receptors include tumor necrosis factor receptor 1 (TNFR1), Fas, DR4, and DR5. Studies have shown that DOX upregulates the expression of TNFR1 and Fas in cardiomyocytes and renal tubular epithelial cells. Receptor activation initiates the extrinsic apoptotic cascade, characterized by activation of caspase-8 and caspase-3, along with a shift in the Bcl-2/Bax ratio toward apoptosis (131, 134, 135).

### 3.5 Other molecular mechanisms

TGF-β is a potent inducer of epithelial-mesenchymal transition (EMT) and a central mediator of fibrotic progression in development, tissue repair, and disease (136). Its canonical signaling is mediated by intracellular SMAD transcription factors (137). Upon binding to its receptors (TβRI/TβRII), TGF-β induces phosphorylation of SMAD2/3. The phosphorylated SMAD2/3 complex then associates with SMAD4 to form a trimeric complex, which translocates to the nucleus and promotes transcription of genes involved in EMT and myofibroblast activation (138). ROS, as a key activator of this pathway, not only induces direct cellular injury but also enhances TGF-β signaling, forming a positive feedback loop that accelerates fibrotic progression (139, 140). In anthracycline-treated models, this interplay promotes excessive extracellular matrix (ECM) deposition in cardiac and renal tissues, resulting in myocardial and glomerular sclerosis, interstitial fibrosis, and subsequent organ dysfunction (141–143). Taking DOX as an example, previous experimental studies have confirmed that DOX can upregulate TGF-β expression and activity in cardiac and renal tissues, increase collagen deposition, and elevate the expression of SMAD3 and MMPs (61, 144–146). Collectively, these studies demonstrate that anthracyclines promote cardiorenal injury through activation of the TGF-β/SMAD signaling pathway.

Non-coding RNAs (ncRNAs), including microRNAs (miRNAs), long non-coding RNAs (lncRNAs), and circular RNAs (circRNAs), are a class of functional RNA molecules that regulate gene expression and cellular processes without being translated into protein (147). Among these, miRNAs have been most extensively studied in disease pathogenesis due to their small size and high target specificity. Growing evidence implicates specific miRNAs in anthracycline-induced cardiorenal toxicity. In anthracycline-induced cardiorenal injury models, studies have revealed characteristic alterations in miRNA expression profiles. For instance, anthracyclines upregulate pro-oxidative miRNAs such as miR-140-5p and miR-34a in cardiomyocytes and elevate pro-apoptotic miRNAs, including miR-874-3p and miR-466o-3p, in renal tubular epithelial cells (148–151). By targeting key molecules involved in apoptosis, inflammation, oxidative stress, and fibrosis, these dysregulated miRNAs synergistically promote tissue injury and functional decline in both the heart and kidney (152). This post-transcriptional regulatory mechanism suggests that anthracyclines disrupt not only protein-coding genes but also the complex regulatory RNA networks that maintain cellular homeostasis.

## 4 Risk factors

Anthracycline-induced cardiorenal toxicity results from the interaction of multiple risk factors, many of which are closely associated with the underlying pathophysiology of the heart and kidneys (153). Among pre-existing conditions, metabolic disorders such as hypertension, diabetes, and obesity are major clinical determinants that increase susceptibility to this toxicity (154).

Hypertension imposes a dual burden, acting as both a risk factor and a direct driver of cardiorenal injury. Elevated blood pressure increases cardiac afterload and intraglomerular pressure, promoting left ventricular hypertrophy and myocardial fibrosis over time and ultimately impairing both diastolic and systolic cardiac function. In the kidneys, sustained elevation in intraglomerular pressure accelerates glomerulosclerosis, damages the filtration barrier, and leads to proteinuria (155). Additionally, chronic hypertension activates RAAS and NADPH oxidase, thereby increasing ROS production and promoting oxidative stress. It also damages the vascular endothelium, activates immune signaling pathways, and promotes the release of inflammatory cytokines such as TNF-α and IL-6 via NF-κB activation (156, 157). When anthracyclines are administered, their inherent ability to generate ROS and activate inflammatory signaling synergizes with hypertensive pathology, further amplifying oxidative stress and inflammatory responses. Disordered glucose metabolism, particularly diabetes and insulin resistance, represents another risk factor exacerbating anthracycline-induced cardiorenal toxicity (158). Insulin resistance impairs myocardial glucose uptake, thereby restricting the heart's primary energy supply. Anthracyclines further exacerbate this energy deficit by damaging mitochondria and inhibiting respiratory chain activity. In the kidneys, insulin resistance enhances glucose reabsorption via sodium-glucose cotransporter 2 (SGLT2) in proximal tubules, thereby reducing urinary glucose excretion (159). At the same time, the hyperglycemic milieu further intensifies anthracycline-induced oxidative stress, accelerating the formation of advanced glycation end products (AGEs). Upon binding to their receptors (RAGE), AGEs activate inflammatory signaling pathways, thereby aggravating myocardial mitochondrial damage and renal interstitial fibrosis (160).

Dose dependency is a hallmark of anthracycline-induced cardiorenal injury, with cumulative exposure serving as a key determinant of risk. However, the incidence of toxicity varies considerably depending on treatment duration and individual comorbidities. The association between cumulative anthracycline dose and cardiac dysfunction has been well established. Available data indicate a clear association between anthracycline therapy and left ventricular dysfunction, reduced ejection fraction, and symptomatic heart failure, with an overall incidence of approximately 5% (161). Specifically, at moderate cumulative doses (250–400 mg/m^2^), 25%–35% of patients develop asymptomatic left ventricular dysfunction. At a cumulative dose of approximately 400 mg/m^2^, the incidence of congestive heart failure is around 3.5%. This risk increases sharply at higher cumulative exposures, reaching 7%–16% at 550 mg/m^2^ and 18%–48% at 700 mg/m^2^ (59). Limiting the cumulative anthracycline dose therefore remains a key strategy to reduce the risk of heart failure. Anthracycline-induced nephrotoxicity also exhibits dose dependency, although its clinical manifestation is influenced by concomitant medications and baseline renal function. In combination chemotherapy regimens, particular attention should be paid to potential additive or synergistic nephrotoxicity when anthracyclines are used with other agents, such as alkylating or platinum-based drugs. In this context, careful control of anthracycline dosing, together with optimization of concomitant therapies, is essential to minimize compounded renal injury. Furthermore, baseline renal function is a key determinant of the dose-toxicity relationship. In individuals with preserved renal function, efficient drug clearance may mitigate apparent dose-dependent nephrotoxicity. In contrast, patients with pre-existing renal impairment have reduced excretory capacity, leading to increased drug accumulation at equivalent doses and a more pronounced nephrotoxic effect (162).

**Figure 4:**
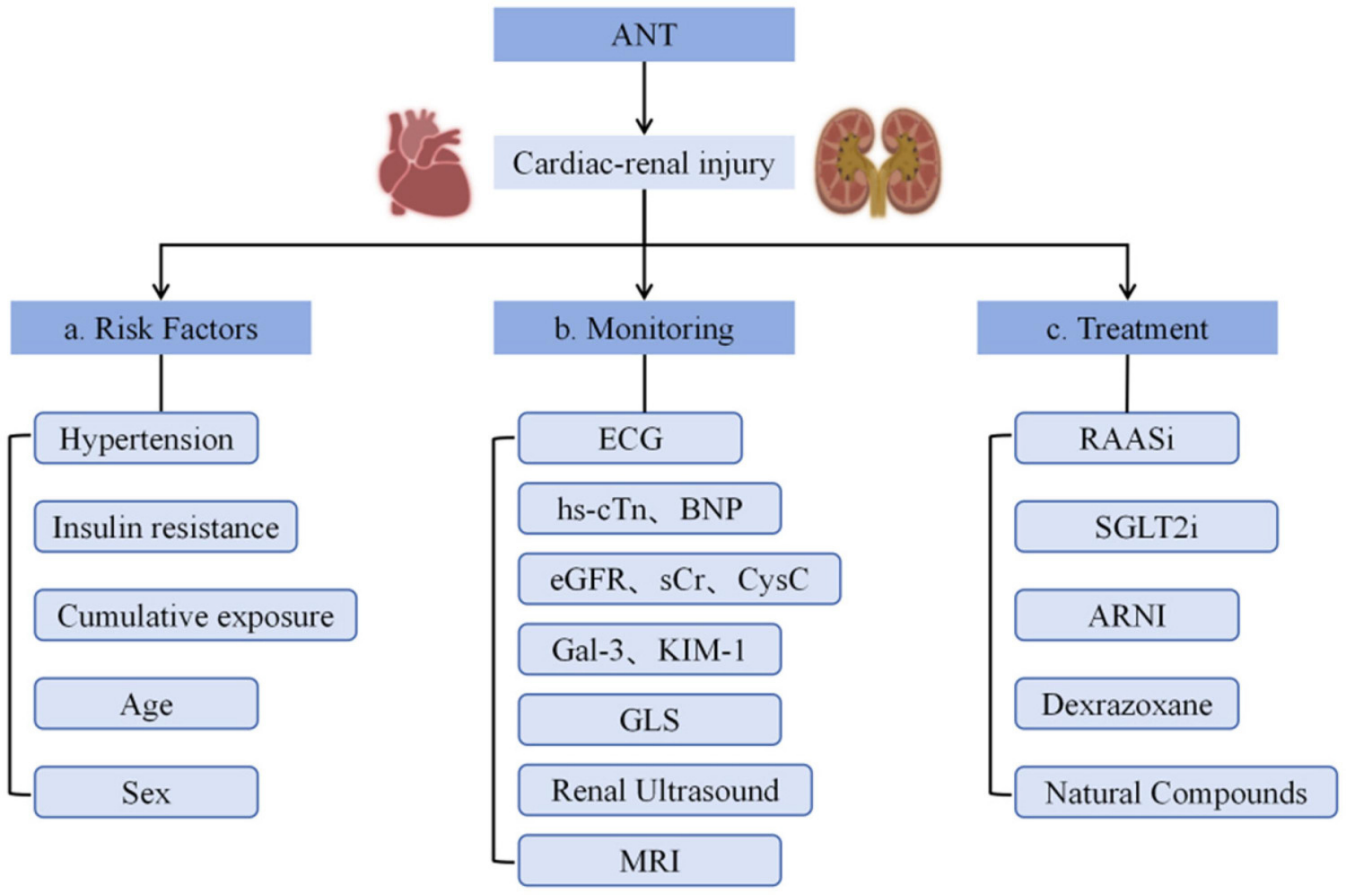
Summary of risk factors, monitoring, and treatment strategies for anthracycline-induced cardiorenal toxicity. ANT, anthracyclines; ECG, electrocardiography; GLS, global longitudinal strain; MRI, magnetic resonance imaging; RAASi, renin-angiotensin-aldosterone system inhibitors; SGLT2i, sodium-glucose cotransporter 2 inhibitors; ARNI, angiotensin receptor-neprilysin inhibitor.

In addition, age and sex are important risk factors for anthracycline-induced cardiorenal toxicity. Owing to incomplete physiological development or age-related functional decline, children and older adults exhibit increased susceptibility to anthracycline-induced cardiorenal toxicity and may experience severe adverse effects even at relatively low doses (7). Sex-based differences in risk have also been observed. Clinical data indicate that prepubertal girls and postmenopausal women generally have a higher risk of anthracycline-related toxicity compared with age-matched males, whereas premenopausal women appear relatively protected (163). This pattern suggests a cardiorenal-protective role for estrogen, potentially mediated by its modulation of oxidative stress pathways and mitochondrial function (164). Recognizing these risk profiles is crucial for developing personalized monitoring strategies and implementing preventive measures in high-risk patients (Figure 4).

## 5 Monitoring

Early detection through systematic monitoring is essential for the prevention and mitigation of anthracycline-induced cardiorenal injury. This approach requires the integration of several complementary modalities, including electrocardiography (ECG), biomarkers, and advanced imaging techniques. Together, these tools enable clinicians to detect characteristic structural and functional alterations before they progress to irreversible organ damage (Figure 4) (153).

### 5.1 Electrocardiogram

The ECG remains a first-line tool for monitoring anthracycline therapy, valued for its accessibility, rapidity, and non-invasive nature. Early signs of cardiac injury, such as ST-T segment deviations or QT-interval prolongation, can appear on the ECG before symptoms emerge. Current guidelines recommend obtaining a baseline ECG prior to initiating therapy, providing a reference point for tracking changes throughout the chemotherapy course (5). Nevertheless, the ECG has clear limitations. While useful for identifying overt, symptomatic cardiotoxicity, it lacks sufficient sensitivity to reliably detect subclinical myocardial injury. Relying solely on ECG may therefore miss the optimal window for early intervention. In clinical management, it is best viewed as a foundational element of a broader monitoring strategy that incorporates more sensitive modalities.

### 5.2 Biomarkers

Circulating biomarkers provide a dynamic, molecular-level assessment of cardiorenal stress, offering valuable complementary information to imaging and functional assessments. High-sensitivity cardiac troponins (hs-cTn) play a central role in this approach. Beyond their established role in diagnosing acute coronary syndromes, elevated hs-cTn levels indicate myocardial injury even in non-ischemic conditions such as acute heart failure, where they are strongly associated with increased mortality risk (29). In patients with CKD, hs-cTn levels are inversely correlated with estimated GFR and serve as a powerful tool for cardiovascular risk stratification (165). In the setting of cardiorenal dysfunction, hs-cTn can help assess disease severity and prognosis; however, levels should be interpreted in the context of renal function to avoid false-positive results due to reduced clearance. Natriuretic peptides, including BNP, reflect ventricular wall stress and volume overload. Plasma BNP concentrations increase in heart failure and, independently, in renal dysfunction even in the absence of overt cardiac disease (166). However, in renal failure, BNP levels may be elevated due to reduced clearance rather than cardiac strain, necessitating careful interpretation alongside other clinical indicators (167). Serum creatinine (sCr) and proteinuria remain cornerstone indicators for diagnosing and staging CKD and for identifying renal impairment in patients with heart failure. However, sCr has low sensitivity and specificity for early AKI and is influenced by age, sex, diet, and body composition (168). Compared with sCr, serum cystatin C is less influenced by these factors and has emerged as a more robust independent predictor of all-cause mortality in patients with AKI or cardiovascular disease (29). Other promising biomarkers include galectin-3 and KIM-1, which demonstrate relatively high sensitivity and specificity for renal injury but have not yet been validated for routine clinical use (168).

### 5.3 Imaging examinations

Modern imaging extends monitoring beyond functional assessment to include tissue characterization and hemodynamic evaluation. In echocardiography, conventional LVEF remains a standard metric, but its variability and insensitivity to early changes are well recognized. Global longitudinal strain (GLS), derived from speckle-tracking echocardiography, quantitatively assesses myocardial contractility and can detect subclinical systolic dysfunction before significant changes in LVEF occur (169). This makes GLS a valuable tool for early risk stratification in patients receiving anthracyclines (170). However, standardization remains challenging due to variability in strain measurements across different ultrasound systems. To ensure consistency, serial assessments should ideally be performed using the same vendor platform before and after chemotherapy (171). Doppler assessment of intrarenal venous flow provides insight into renal congestion, correlating with right-atrial pressure and offering prognostic value in heart failure (23). Although parameters such as the renal resistive index (RRI) are not yet validated for routine clinical use, integrating renal ultrasonography—including assessment of renal structure, echogenicity, and cortical thickness—can support a more comprehensive evaluation of cardiorenal status (165).

As imaging technology advances toward quantitative and multiparametric tissue characterization, magnetic resonance imaging (MRI) enables high-resolution, non-invasive assessment of cardiac and renal structure and function, providing a valuable complement for the early detection of subclinical lesions. Cardiac magnetic resonance imaging (CMR) is the non-invasive reference standard for quantifying ventricular volume, function, and tissue composition. Its ability to identify myocardial edema, fibrosis, and subtle structural alterations makes it particularly well-suited for detecting anthracycline-induced injury at the subclinical stage, serving as a crucial complement to conventional echocardiography (153). Renal magnetic resonance imaging enables multidimensional assessment of renal structure, blood flow, tissue fibrosis, and metabolic status, reflecting both injury and functional reserve. Its key advantage lies in the non-invasive, contrast-free quantification of hemodynamic parameters such as renal blood flow and filtration rate, independent of baseline renal function, making it a powerful tool for the subclinical monitoring of kidney injury (172).

## 6 Treatment strategies

To mitigate anthracycline-induced cardiorenal toxicity, therapeutic strategies are increasingly focused on targeting both organs concurrently. The goal is to interrupt the vicious cycle of mutual injury by tackling shared pathological processes—oxidative stress, inflammation, mitochondrial dysfunction, apoptosis, and fibrosis (Figure 4).

### 6.1 RAAS inhibitors

RAAS inhibitors are cornerstone therapies for hypertension, heart failure, and CKD. Clinical evidence indicates that ACEIs and angiotensin II receptor blockers (ARBs) provide significant cardiorenal protective effects (173). In preclinical models, telmisartan (an ARB) and captopril (an ACEI) demonstrate significant protection against DOX-induced injury. These agents reduce circulating biomarkers of myocardial injury and renal dysfunction, including cardiac enzymes and renal function indices. They also attenuate histological alterations, including myocardial interstitial edema, fibrosis, and tubular necrosis, and restore redox balance by decreasing tissue levels of MDA, iNOS, and NO while increasing GSH levels and the activity of antioxidant enzymes such as GPx and SOD (42). Notably, RAAS inhibitors may induce hyperkalemia, which is associated with increased risks of cardiovascular events, hospitalization, and mortality. Studies indicate that co-administration of sodium-glucose cotransporter 2 inhibitors (SGLT2i) with RAAS inhibitors can alleviate this electrolyte disturbance while further enhancing overall cardiorenal protection (174). These findings position RAAS inhibition as a foundational strategy in mitigating anthracycline-induced cardiorenal toxicity.

### 6.2 SGLT2i

SGLT2i (e.g., dapagliflozin, empagliflozin) reduce the risk of adverse events and all-cause mortality in patients with kidney disease and heart failure. Their mechanisms include improving renal hemodynamics, modulating vasoactive mediators, and attenuating oxidative stress, thereby establishing them as key therapeutic agents in cardiorenal medicine (175). In the kidney, SGLT2i can reduce glucose and sodium reabsorption in the proximal tubule. This reduces intraglomerular stress, improves renal cortical oxygenation, and preserves tubular function, while also decreasing the incidence of anemia and hyperkalemia. In the heart, SGLT2i reduce volume overload through osmotic diuresis and natriuresis, improve myocardial energetics by promoting ketone body utilization, and suppress sympathetic overactivity and NLRP3 inflammasome activation, thereby attenuating inflammation and fibrosis (176). Accumulating clinical evidence indicates that SGLT2i confer significant cardiorenal protective effects during anthracycline therapy (177). Preclinical studies have further elucidated the mechanisms underlying these benefits. SGLT2i attenuate anthracycline-induced myocardial fibrosis through anti-inflammatory, antioxidant, and anti-apoptotic effects, while downregulating the expression of pro-inflammatory cytokines and markers of myocardial injury, including high-sensitivity C-reactive protein (47, 178, 179).

### 6.3 Angiotensin receptor–neprilysin inhibitors

Sacubitril/valsartan—an angiotensin receptor–neprilysin inhibitor (ARNI)—is approved for the treatment of heart failure with reduced ejection fraction. By simultaneously enhancing natriuretic peptide activity through neprilysin inhibition and blocking angiotensin II receptors, it provides dual-pathway modulation that confers integrated cardiorenal protection, particularly in patients with concomitant cardiac and renal disease (180). Clinically, sacubitril/valsartan reduces the composite risk of adverse cardiovascular events and progression to end-stage renal disease, slows the decline in estimated GFR, and is associated with a favorable safety profile, with low discontinuation rates due to renal adverse events (181). In patients receiving breast cancer chemotherapy, this agent has been shown to mitigate cardiotoxicity by lowering NT-proBNP levels, alleviating mitral regurgitation, and improving diastolic function without significantly affecting serum creatinine or potassium levels (182). Preclinical studies further attribute its protective effects against DOX-induced cardiomyopathy to antiarrhythmic, anti-inflammatory, antioxidant, and anti-apoptotic properties (183). Although these findings are promising, large-scale clinical trials specifically evaluating ARNI in anthracycline-induced cardiorenal toxicity are still lacking, highlighting an important gap in the current evidence base.

### 6.4 Dexrazoxane

Dexrazoxane is the only drug approved by the U.S. Food and Drug Administration (FDA) for the prevention of anthracycline-induced cardiotoxicity. Extensive clinical evidence demonstrates that dexrazoxane significantly reduces cardiovascular adverse events in patients receiving anthracycline chemotherapy (184). Preclinical studies suggest that this protective effect may extend to the kidneys, as dexrazoxane mitigates pathological injury and apoptosis in both cardiac and renal tissues. Its protective effects are associated with modulation of key oxidative stress–related enzymes, resulting in reduced myocardial and renal interstitial fibrosis (10, 185). However, large-scale clinical studies validating its renoprotective effects are currently lacking. Furthermore, the 2022 ESC Guidelines do not recommend the routine use of dexrazoxane in patients receiving anthracycline therapy. Its use is recommended only for high-risk patients, such as those with baseline LVEF <50%, age ≥80 years, or an anticipated cumulative DOX dose ≥250 mg/m^2^ (186).

### 6.5 Natural compounds

In recent years, oncology consensus statements worldwide have emphasized the importance of implementing cardioprotective strategies during cancer therapy. In the context of anthracycline-induced cardiorenal injury, identifying effective interventions is particularly important. Among available strategies, natural compounds have attracted considerable attention due to their multi-target pharmacological properties. Accumulating evidence suggests that these agents modulate key pathological processes—including oxidative stress, inflammatory responses, mitochondrial dysfunction, and apoptosis—thereby interrupting the vicious cycle of cardiorenal interaction and conferring both cardioprotective and renoprotective effects (187). To systematically appraise the current evidence, we conducted a literature search of the PubMed and Web of Science databases covering the past decade using the following keywords: “anthracyclines”, “cardiotoxicity”, “nephrotoxicity”, “natural compounds”, and “doxorubicin.” Only English-language publications were included. Based on the level of evidence, the identified natural compounds were categorized into two groups: those evaluated in clinical studies and those supported solely by preclinical evidence.

#### 6.5.1 Clinical studies

Currently, several natural products have been evaluated in clinical studies and have demonstrated potential cardioprotective effects. Crocin, a principal bioactive constituent of *Crocus sativus L*., exhibits antioxidant, anti-inflammatory, and mitochondria-regulating properties (188, 189). A multicenter randomized controlled trial conducted by Xing et al. evaluated the cardioprotective effects of crocin in breast cancer patients receiving anthracycline-based chemotherapy. The results showed that crocin administration significantly reduced the incidence of LVEF decline, suggesting its potential as a safe and effective cardioprotective agent (190). Nigella sativa oil, extracted from its seeds, contains thymoquinone, a compound with well-documented antioxidant and anti-inflammatory activities (191). Clinical studies have shown that, in pediatric patients with acute lymphoblastic leukemia undergoing DOX-based chemotherapy, adjunctive treatment with Nigella sativa oil attenuates cardiotoxicity and improves systolic cardiac function (192). In addition, traditional Chinese medicines such as *Salvia miltiorrhiza and Astragalus membranaceus* are widely used in clinical practice to improve cardiac function and reduce biomarkers of myocardial injury. However, high-quality randomized controlled trials specifically targeting anthracycline-induced cardiorenal toxicity remain limited, warranting further investigation.

#### 6.5.2 Preclinical studies

Compared with clinical evidence, most data on the protective effects of natural compounds against anthracycline-induced cardiorenal toxicity are derived from preclinical studies. A common feature of these compounds is their ability to modulate oxidative stress, inflammatory responses, and cell death pathways.

With respect to renal injury, plant extracts and flavonoids have demonstrated significant protective effects. Leaf extracts of *Asparagus falcatus L.*, enriched in steroidal saponins, polyphenols, and flavonoids, significantly improve renal function and ameliorate histopathological damage in DOX-induced nephrotoxicity models, primarily by enhancing antioxidant defenses and suppressing inflammation and apoptosis (193). Apigenin, a representative natural flavonoid, ameliorates DOX-induced renal injury by modulating NF-κB, MAPK, and PI3K/Akt signaling pathways and activating the Nrf2/HO-1 antioxidant axis, thereby reducing oxidative stress and inflammatory responses (194). Consistently, Gao et al. demonstrate that apigenin reduces MDA levels, increases SOD and GSH activity, and downregulates inflammatory factors such as TNF-α and IL-1β, resulting in improved renal function and attenuated tissue injury *in vivo* (70).

Regarding cardiotoxicity, natural compounds such as ginsenosides have demonstrated beneficial effects on myocardial injury, hypertrophy, and fibrosis (195). Mechanistically, ginsenoside Rb1 ameliorates mitochondrial dysfunction by regulating autophagy and ferroptosis and attenuates pathological cardiac remodeling via inhibition of the CaN/NFATc4/GATA4 pathway (196, 197). Ginsenoside Rh2 ameliorates cardiac injury and remodeling by modulating the Bax/Bcl-2 balance and suppressing inflammation and fibrosis without compromising antitumor efficacy (198). Ginsenoside F1 exerts antioxidant and anti-apoptotic effects through activation of the Nrf2 and AKT/Bcl-2 signaling pathways (199). Collectively, ginsenosides appear to confer cardioprotection through the coordinated regulation of mitochondrial homeostasis, ferroptosis, and inflammation.

In addition, several terpenoids and polyphenols exhibit dual cardiorenal protective effects. Costunolide (COS) exerts antioxidant and anti-inflammatory effects by activating the Nrf2/Keap1 pathway and suppressing the NF-κB/NLRP3 axis (200, 201), thereby significantly reducing cardiorenal injury markers and inflammatory cytokine levels in DOX-treated models (58). Tanshinone IIA exhibits anti-inflammatory and antioxidant properties and attenuates DOX-induced injury through modulation of the PTEN/AKT pathway, while also enhancing chemosensitivity (202, 203). Anethole, a major component of essential oils, possesses antioxidant, anti-inflammatory, and anticancer properties (204). In DOX-induced models of cardiac and renal injury, anethole alleviates inflammation and apoptosis by inhibiting the TLR4/MyD88/NF-κB signaling pathway and modulating the Bcl-2/Bax balance, thereby significantly reducing cardiac and renal injury markers (205). Quercetin, a representative polyphenolic compound, mitigates DOX-induced cardiorenal injury by preserving mitochondrial function, suppressing inflammation, and inhibiting apoptosis and may also enhance the efficacy of chemotherapy (206). Crocin has also demonstrated multi-organ protective effects in preclinical studies, improving cardiac and renal function and attenuating tissue injury through regulation of oxidative stress and NF-κB–mediated inflammatory signaling (64, 207).

Emerging evidence further suggests that several natural compounds exert protective effects through the regulation of ferroptosis and Nrf2-associated signaling pathways. Protosappanin A attenuates DOX-induced myocardial injury by targeting the ACSL4/FTH1 axis and suppressing ferroptosis (208). Glycyrrhetinic acid improves cardiac function via activation of the Nrf2/HO-1 pathway, thereby reducing oxidative stress and apoptosis (209). In addition, Tripterygium glycoside fraction N2 (TG-n2) and Astragaloside IV show potential in ameliorating DOX-induced renal injury by suppressing apoptosis and modulating ferroptosis, respectively (71, 134) (Table 1).

**Table 1:** Representative natural compounds for treating anthracycline-induced cardiorenal toxicity.

Compound	Model	Concentration	Duration	Mechanisms	Ref.
*Asparagus falcatus L.*	Rats of nephrotoxicity	200 mg/kg/day	28 days	Antioxidant, Anti-inflammatory, Antiapoptosis	(193)
Apigenin	Mice of nephrotoxicity	500 mg/kg/day	2 weeks	Antioxidant, Anti-inflammatory	(70)
Ginsenoside Rb1	Mice of cardiotoxicity	40 mg/kg/day	7 days	Inhibition of autophagy and ferroptosis	(196)
Ginsenoside Rh2	Mice of cardiotoxicity	20 mg/kg/day	3 weeks	Antiapoptosis, Anti-inflammatory	(198)
Ginsenoside F1	Rats of cardiotoxicity	50 mg/kg/day	7 weeks	Antiapoptosis, Antioxidant	(199)
Costunolide	Rats	50 mg/kg/day	4 weeks	Antiapoptosis, Antioxidant, Anti-inflammatory	(58)
Anethole	Rats	250 mg/kg/day	15 days	Antiapoptosis, Antioxidant, Anti-inflammatory	(205)
Crocin	Rats of cardiotoxicity	40 mg/kg/day	15 days	Antiapoptosis, Antioxidant, Anti-inflammatory	(207)
	Rats of nephrotoxicity	100 mg/kg/day	3 weeks	Antioxidant, Anti-inflammatory	(64)
Tanshinone IIA	Mice	10 mg/kg/48h	5 days	Antiapoptosis	(203)
Quercetin	Rats of cardiotoxicity	50 mg/kg/day	11 days	Antiapoptosis, Antioxidant, Improve mitochondria	(210)
	Rats of nephrotoxicity	50 mg/kg/day	15 days	Antioxidant, Anti-inflammatory	(211)
Protosappanin A	Mice of cardiotoxicity	5/20 mg/kg	48 h	Inhibition of ferroptosis	(208)
Glycyrrhetinic acid	Mice of cardiotoxicity	50 mg/kg/day	28 days	Antiapoptosis, Antioxidant, Improve mitochondria	(209)
TG-n2	Rats of nephrotoxicity	10 mg/kg/day	4 weeks	Antiapoptosis	(134)
Astragaloside IV	Rats of nephrotoxicity	10 mg/kg/day	5 weeks	Inhibition of ferroptosis	(71)

Overall, natural compounds exhibit multi-target and multi-pathway advantages in mitigating anthracycline-induced cardiorenal toxicity. However, the current evidence remains largely preclinical, with limited clinical validation. Future studies should focus on high-quality randomized controlled trials and alignment with authoritative consensus recommendations to further clarify their efficacy and safety and to facilitate clinical translation.

## 7 Conclusion

Anthracycline-induced cardiorenal toxicity represents a complex clinical challenge driven by interconnected pathological cascades. RAAS serves as a central signaling axis coordinating crosstalk between the heart and kidneys. Injury in both organs shares common molecular drivers: ROS and inflammatory mediators generated during oxidative stress and immune activation exert systemic effects, while mitochondrial dysfunction impairs cellular energetics, leading to parallel functional decline. Although substantial progress has been made in deciphering these mechanisms, the precise temporal sequence and reciprocal nature of cardiorenal injury remain to be fully defined.

Electrocardiography, circulating biomarkers, and imaging modalities constitute the core diagnostic tools; however, evaluation of either organ in isolation struggles to capture their dynamic interdependence. Therefore, it is essential to establish an integrated framework for combined cardiorenal monitoring and risk stratification, particularly in high-risk populations such as older adults, patients with comorbidities, or those receiving high cumulative anthracycline doses. Implementing a structured surveillance strategy spanning baseline assessment, on-treatment monitoring, and post-therapy follow-up is essential. Within this framework, combining sensitive indicators such as hs-cTn with BNP, and GLS with RRI, may facilitate earlier detection of subclinical injury. Therapeutic options remain limited. Dexrazoxane, approved for cardioprotection via iron chelation and antioxidant effects, shows uncertain renal benefit and is associated with adverse effects such as myelosuppression, which limits its routine use. Natural compounds, despite demonstrating promising multi-target effects in preclinical studies, still lack sufficient clinical evidence regarding efficacy, optimal dosing, long-term safety, and interactions with chemotherapy.

In the future, conducting in-depth research into the mechanisms underlying anthracycline-associated cardiotoxicity and nephrotoxicity, with a particular focus on elucidating their mutual detrimental effect, while simultaneously developing personalized risk prediction models, standardizing monitoring protocols, and advancing the clinical translation of natural compounds, will facilitate the early identification and intervention of toxicity. This may help promptly break the vicious cycle of cardiac and renal injury, thereby providing optimal strategies for balancing the therapeutic benefits of anthracyclines with long-term patient safety.
